# Patient and healthcare professional experiences of the Salford Lung Studies: qualitative insights for future effectiveness trials

**DOI:** 10.1186/s13063-020-04655-x

**Published:** 2020-09-17

**Authors:** Kim Gemzoe, Rebecca Crawford, Ann Caress, Sheila McCorkindale, Rebecca Conroy, Susan Collier, Lynda Doward, Renu M. Vekaria, Sally Worsley, David A. Leather, Elaine Irving

**Affiliations:** 1 Real World Study Delivery, Value Evidence and Outcomes, GlaxoSmithKline plc., Research & Development Ltd., Stockley Park West, 1–3 Ironbridge Road, Uxbridge, Middlesex UB11 1BT UK; 2 RTI Health Solutions, Manchester, M20 2LS UK; 3 School of Human and Health Sciences, University of Huddersfield, Huddersfield, HD1 3DH UK; 4 NIHR Clinical Research Network Greater Manchester, Manchester, M13 9NQ UK; 5 UK Medical, GlaxoSmithKline plc, Uxbridge, UB11 1BT UK; 6 Real World Study Delivery, Value Evidence and Outcomes, GlaxoSmithKline plc., Research & Development Ltd., Stevenage, SG1 2NY UK; 7 Global Respiratory Franchise, GlaxoSmithKline plc., Brentford, TW8 9GS UK

**Keywords:** Salford Lung Studies (SLS), Effectiveness trial, Trial experience

## Abstract

**Background:**

Randomized controlled trials (RCTs) conducted in the routine care setting provide the opportunity to better understand the effectiveness of new medicines but can present recruitment difficulties. An improved understanding of the challenges/opportunities for patient and healthcare professional (HCP) engagement in clinical research is needed to enhance participation and trial experience. In this study, we explored patient and HCP drivers for, and experiences of, participation in the Salford Lung Studies (SLS), and their views on future trial participation and the overall value of such trials.

**Methods:**

This was a qualitative study set in Salford, UK, comprising patient telephone interviews (*N* = 10) and HCP advisory boards (one with general practitioners [GPs], one with practice managers [PMs]); all individuals had participated in the SLS. Semi-structured telephone interviews were recorded, transcribed and analysed thematically. Advisory board meetings were analysed based on transcriptions of audio recordings and field notes.

**Results:**

For patients, key positive aspects of the SLS were the ease/convenience of study assessments and excellent relationships with study nurses. GPs and PMs considered the SLS to be well-organized and highlighted the value of research nurse support; they also described minor challenges relating to trial systems, initial financial strain on practices and staff turnover. All participants indicated that they were very likely to participate in future trials, citing a design closely aligned with routine care practice as essential. Several strategies to encourage trial participation were suggested, such as clearly communicating benefits to patients and ensuring flexible study assessments.

**Conclusions:**

Patients and HCPs had positive experiences of the SLS. The study design, closely aligned with routine care, was considered important to their high likelihood of participating in future trials. The experiences of patients and HCPs in the SLS provide valuable insights that will help inform future best practice in the design and conduct of future real-world effectiveness RCTs in primary care. The detailed first-hand experiences of HCPs will be of significant value to others considering engaging in clinical research and participating in effectiveness RCTs.

## Background

Effectiveness trials are randomized controlled trials (RCTs) conducted in clinical settings that more closely reflect everyday clinical practice, in patients that more closely represent those to whom the medicine will be prescribed. Such trials provide the opportunity to complement data collected from traditional efficacy RCTs, where clinical settings are idealized and patient populations are highly selected and often represent a minority of the broader patient population eligible for treatment [1, 2]. However, embedding clinical research into a routine care setting can be challenging with respect to the required infrastructure as well as the impact on treating physicians’ time. Furthermore, patients and physicians may already have access to new medicines, so the drive to participate in clinical studies can be low. Successful recruitment often requires extensive resources and significant engagement from healthcare professionals (HCPs) [3].

Previous studies have shown that patient participation in clinical research is associated with factors such as altruism, advancing research, access to new medicines, understanding their own condition, patient circumstances and study design [4–7]. Compared with traditional efficacy trials, effectiveness studies may be less burdensome for patients, making it easier for them to participate. There may be common drivers for patient participation in effectiveness trials and efficacy trials, however, to our knowledge, this has not been confirmed previously.

Relatively little is known about the experiences and engagement of HCPs in clinical research. While physicians may initially express an interest in participating in clinical studies, translation of this interest into actual recruitment of patients may be low [8–10]. Recognized barriers to general practitioner (GP) involvement in clinical studies include resource, physical space, facilities and time requirements, insufficient interest in the research question and disruption to normal clinical practice [11–14]. For patients, a lack of awareness about how clinical research can improve care often results in reluctance, indecision or refusal to participate in clinical trials [15–17]. These challenges underscore the need to better understand the drivers of patient and HCP engagement in clinical research, particularly in the context of effectiveness RCTs.

The Salford Lung Studies (SLS) in chronic obstructive pulmonary disease (COPD) and asthma were world-first, phase III RCTs that evaluated the clinical effectiveness and safety of a pre-licensed medication (fluticasone furoate/vilanterol) in a large population of UK primary care patients [3, 18–21]. To increase engagement of physicians and patients, the SLS team focused on ensuring that the SLS trial design was aligned as far as possible to routine care, with limited study-specific visits, facilitating data collection by providing nurse support for data entry, and enrolling patients at primary care practices with integrated care records to limit the impact on physician time [3]. Patients were managed by their usual GPs and collected study medications where possible from their local participating community pharmacy. All patients received free respiratory prescriptions on-study.

This study used the unique and valuable opportunity provided by the SLS to explore patient and HCPs’ perceptions of the value of this type of research, their experiences during the different trial stages and their views on how participation in future effectiveness trials might be improved. This work is of interest to anyone considering engaging with clinical research and participating in effectiveness RCTs and will inform stakeholders looking to improve the design and conduct of future real-world effectiveness studies in primary care.

## Methods

### Study design and participants

This qualitative study used a qualitative description approach to explore patient and HCPs’ (GPs and practice managers [PMs]) experiences of participation in the COPD and asthma SLS [3, 18–21]. Qualitative description is particularly valuable where the intention is to collect information on individuals’ experiences in relation to a specific topic and based on what they say [22, 23]. Telephone interviews with patients and advisory board meetings with HCPs in Salford, UK, were conducted to collect data on four key topics: pre-trial experience, experience during the trial, post-trial experience and future trial experience. Interviews/advisory boards were facilitated by an independent not-for-profit research organization (RTI Health Solutions [RTI-HS], Manchester, UK) in collaboration with the study sponsor (GlaxoSmithKline plc.). RTI-HS and its researchers, who had between 10 and 35 years of research experience, had no relationships with participants and no vested interest in the outcome of the study.

**Fig. 1:**
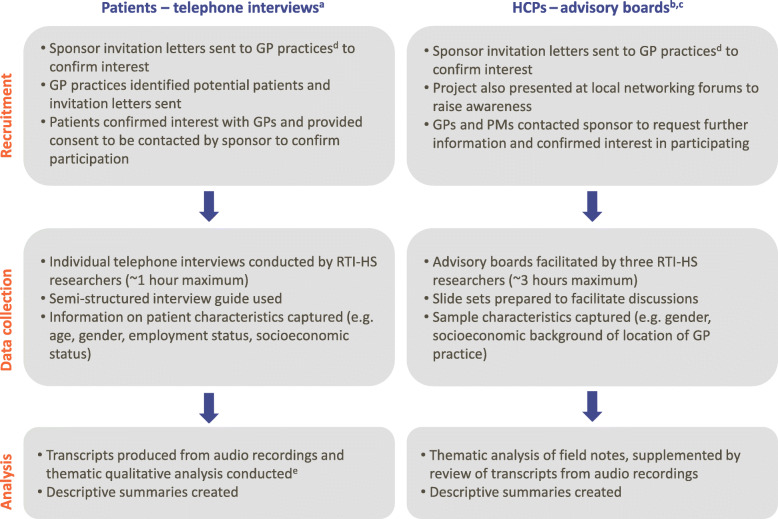
Participant recruitment, data collection and analysis processes. ^a^Patients were recruited through purposive sampling: GPs that had participated in the SLS and who had indicated their intention to participate in the qualitative study identified SLS patients and sent them a patient-centred invitation letter (provided by the sponsor). ^b^GPs and PMs were recruited through opportunistic sampling: the sponsor sent an invitation letter detailing the qualitative study objectives and information about the advisory boards to all GP sites that had participated in the SLS. ^c^A single 1-h telephone interview was conducted with a PM who was unable to attend the PM advisory board; this interview was facilitated by an interview guide that had been developed based on the PM version of the advisory board slide set. ^d^The sample of GP practices contacted was selected to cover a broad range of socioeconomic spread within Salford surrounding areas. ^e^Analysis conducted using the ATLAS.ti software (v7.5). Abbreviations: GP, general practitioner; HCP, healthcare professional; PM, practice manager; RTI-HS, RTI Health Solutions; SLS, Salford Lung Studies

Figure 1 summarizes the participant recruitment process. Opportunistic and purposive sampling was used to recruit HCPs and patients, respectively. Invitation letters were sent by the sponsor to GP practices that had participated in the SLS, providing details of the study and inviting GPs and PMs to take part in the advisory boards. The local Clinical Research Network provided additional support in identifying and contacting PMs. The practice sample was selected to cover a broad range of socioeconomic areas within Salford and Greater Manchester based on the English Index of Multiple Deprivation [24]. GPs who confirmed their interest then identified and contacted potential patients who had participated in the SLS. All participants were reimbursed at fair market value for their time and expenses during the study.

### Data collection

Data collection methods are summarized in Fig. 1. Semi-structured telephone interviews with patients, supported by an interview guide focused around the four key topics (Table 1), were conducted by two experienced, independent RTI-HS researchers. Interviews lasted approximately 1 h and captured patients’ views and experiences of the trial process and their suggestions for increasing participation and improving the patient experience. Patient characteristics were collected prior to the start of the interview. Interviews were audio-recorded with patients’ permission and later transcribed verbatim and de-identified by a medical transcription vendor (Accuro, Knutsford, UK).

**Table 1:** Topics and themes for discussion in patient telephone interviews and HCP advisory boards

Topic	Patient telephone interviews (interview guide)^**a**^	HCP advisory boards (slide sets)^**b**^
**Pre-trial experience**	• Invitation to participate • Reasons for participation • Information about the trial • Previous research experience	• Reasons for participation^c^ • Perceived value of participation^c^ • Positive/negative experiences^c^ • Challenges and solutions^c^ • Engagement of the GP practice and PMs in SLS^d^ • PM views on GP’s perceived value of SLS^d^ • Trial preparation, challenges and solutions^d^ • Suggestions for improvements
**Experience during the trial**	• Positive/negative experiences • Impact of experience	• Positive/negative experiences^c^ • Expectations of the trial^c^ • Operational impact of SLS^d^ • Support received for managing the trial^d^ • Challenges and solutions • Suggestions for improvement
**Post-trial experience**	• Perceived value of SLS • Trial results • Missing aspects from SLS	• Views on the process used to share trial results with GPs^c^ • Perceived value of plain language summary to patients^d^ • Suggestions for improvements^d^ • Expectations at end of trial • Views on receipt of results and dissemination to patients
**Future trial experience**	• Likelihood of participating in future trials; reasons for and against • Suggestions to encourage participation or improve patient experience • Views on patient involvement in trial design	• Suggestions to increase GP engagement in future trials^c^ • Perceptions of research after participation in SLS^d^ • Likelihood of participating in future trials • Suggestions to improve trial experience

*GP* general practitioner, *HCP* healthcare professional, *PM* practice manager, *SLS* Salford Lung Studies

^a^The interview guide was focused on four key topics: pre-trial experience, experience during the trial, post-trial experience and future trial experience. Each section included prompting questions to gather information from patients on specific themes/aspects of the SLS experience

^b^The slide sets prepared for the GP and PM advisory boards also comprised four key topics: pre-trial experience, experience during the trial, post-trial experience and future trial experience

^c^Content specific to the advisory board with GPs

^d^Content specific to the advisory board with PMs

Two separate HCP advisory boards (one with GPs, one with PMs, who had participated in the SLS) were held in Salford, UK. Each advisory board lasted approximately 3 h and captured HCPs’ insights on the operational and logistical experiences during the SLS, their perceived value of this type of research and their views on how participation in future trials might be improved. Three experienced, independent, RTI-HS researchers facilitated the advisory boards using pre-prepared slide sets (Table 1). After spontaneous responses were complete, the facilitators used pre-specified prompts to encourage further discussion to ensure that all aspects of the study objectives were explored. RTI-HS researchers are trained in the use of prompts to elicit contributions from advisory board participants without influencing the content of their responses. Discussions were audio-recorded with attendees’ permission and transcribed verbatim by a medical transcription vendor (Accuro, Knutsford, UK). A single telephone interview was held with a PM unable to attend the advisory board; the interview was conducted using a semi-structured interview guide based on the PM advisory board.

### Analysis

Analysis methods are summarized in Fig. 1. Patient transcripts were reviewed by the research team for accuracy and completeness and underwent thematic qualitative analysis using the ATLAS.ti coding software version 7.5 (ATLAS.ti Scientific Software Development GmbH, Berlin, Germany). Each transcript was coded by independent primary and secondary researchers for quality control purposes. The initial coding framework was based on the final interview guide. Development of the coding framework was an iterative process with further themes and codes added to the framework during the analysis in order to capture additional information not included by the codes in the initial framework. Descriptive summaries were produced from extracted excerpts relating to codes within each of the four key topics. Analysis of data from the advisory board discussions with GPs and PMs (and the single telephone interview with a PM) was based on transcripts of the audio-recorded meetings and field notes taken by the two independent researchers facilitating the events who recorded their observations directly into an Excel spreadsheet [25]. The field notes were thematically analysed to identify key themes and this information was supplemented by a review of the meeting transcripts.

## Results

### Participants

Ten patients contacted by participating GPs (*n* = 9) agreed to take part in the telephone interviews. Table 2 presents the characteristics of the 10 patients (three COPD, seven asthma) who participated in the telephone interviews*.* All patients were White, most (7/10) were male and most (7/10) were aged ≥ 55 years.

**Table 2:** Characteristics of SLS patients who participated in the telephone interviews

	Overall (***N*** = 10)	COPD (***n*** = 3)	Asthma (***n*** = 7)
**Gender,** ***n*** **(%)**			
Male/female	7 (70)/3 (30)	2 (66.7)/1 (33.3)	5 (71.4)/2 (28.6)
**Age, years,** ***n*** **(%)**			
18–34	2 (20)	0 (0)	2 (28.6)
35–54	1 (10)	0 (0)	1 (14.3)
55–74	5 (50)	1 (33.3)	4 (57.1)
75+	2 (20)	2 (66.7)	0 (0)
**General health,** ***n*** **(%)**			
Very good	5 (50)	1 (33.3)	4 (57.1)
Good	2 (20)	1 (33.3)	1 (14.3)
Fair	3 (30)	1 (33.3)	2 (28.6)
Poor	0	0 (0)	0 (0)
**Other health conditions,** ***n*** **(%)**			
Yes/no	9 (90)^a^/1 (10)	3 (100)/0 (0)	6 (85.7)/1 (14.3)
**Employment status,** ***n*** **(%)**			
Working full-time/retired	3 (30)/7 (70)	0/3 (100)	3 (42.9)/4 (57.1)
**IMD decile (range)**^**b**^	4–10^c^	9–10	4–10
**Prior research experience,** ***n*** **(%)**			
Yes/no	5 (50)/5 (50)	1 (33.3)/2 (66.7)	4 (57.1)/3 (42.9)

*COPD* chronic obstructive pulmonary disease, *IMD* Index of Multiple Deprivation, *SLS* Salford Lung Studies

^a^Other health conditions included: atrial flutter, benign prostatic hyperplasia, blood pressure difficulties, hay fever, hypertension, hypothyroidism, post-polio syndrome and pulmonary embolism

^b^The IMD ranks every small area in England from 1 (most deprived area) to 32,844 (least deprived area) and categorizes these into 10 equal groups to determine the deprivation deciles [26]; thus, decile 1 represents the most deprived and decile 10 the least deprived

^c^Nine patients resided in areas considered to be amongst the 30% least deprived neighbourhoods in England. One patient resided in an area considered to be amongst the 40% most deprived neighbourhoods in England

Nine GPs attended the GP advisory board: two were based at the same practice, seven were principal investigators and two were sub-investigators during the SLS. Two GPs had practices in the 10% most deprived areas in England and two had practices in the 10% least deprived areas.

Six PMs attended the PM advisory board. With one exception, PMs were responsible for a single GP practice and there was little site overlap between GPs and PMs. All GP practices were in areas amongst the 40% most deprived in England. The single PM interviewed by telephone was from a GP practice in an area amongst the 10–20% most deprived in England.

### Patient experience

Key themes derived from the analysis of patient interviews are summarized in Table 3 (detailed results in Additional Table 1). Results are presented below as detailed narrative descriptions, with the number of patients whose responses were grouped under each theme also provided. In the pre-trial stage, patients’ rationales for participation included: perceived benefit to personal health (6/10) or to others (3/10), supporting medical science (5/10), access to new treatments (4/10) and reimbursement (2/10). Three patients had minor negative comments regarding the content or length of the study information, but overall, patients regarded the study information as clear and easy to understand:

It was quite explicit; there was a lot of information, but then I think with anything like that, there has to be a lot of information because you have to make informed choices. (Participant 001, female, age range 55–64, asthma)

Patient-reported experience during the trial was largely positive; half of patients had no negative experiences. Patients highlighted the positive relationships with the research nurses (10/10), the convenience (8/10) and ease (7/10) of study assessments and receipt of free prescriptions (2/10):

I think my relationship with the nurse that I saw on a regular basis [was a positive experience]. She was absolutely lovely, and I felt very comfortable talking to her…about my asthma and how I was feeling at the time. (Participant 006, female, age range 45–54, asthma)

It was spread out really easy. Like I said, it wasn’t like it was every week or they were mithering you in your time… It’s hard to turn things down when they make it so easy for you. (Participant 009, male, age range 25–34, asthma)

Only three minor criticisms of the experience during the trial were reported. One patient referred to their local pharmacy not being involved, two patients commented on not being randomized to the new study medication and a further two on not receiving the study results:

I would have liked to have been on the trial meds. I just ended up taking the same inhalers throughout… So I think I would have quite liked to have tried the new one just to see if there was any difference, but that’s part of being in a study isn’t it?... It’s the luck of the draw. (Participant 006, female, age range 45–54, asthma)

My only negative is there being no follow-up to it. It would have been nice if I’d have had a letter from someone on your staff telling me what the results were, how the study was going on… (Participant 002, male, age range 75–84, COPD)

For the post-trial stage, an improved knowledge of COPD and asthma, potential benefits to other people and an interest in receiving the trial results (6/10) were articulated by patients:

For me, it was probably making me consider my asthma symptoms a lot more. I can be quite complacent at times, and think I don’t need to take my inhalers today and I’m feeling OK; and I think I was a bit more on it while I was on the study. (Participant 006, female, age range 45–54, asthma)

All 10 patients reported they would participate in a future study and eight agreed that including patients in the study design phase would be beneficial. Strategies to encourage participation were suggested, such as clearly communicating benefits to patients and flexible study assessments:

You know, make it known, you know, that it is science and that the study is aimed at improving treatments and improving people’s health. (Participant 005, male, age range 65–74, asthma)

Everything we did, it was pretty much at our convenience. So I think that’s quite an important thing because…people have patterns of their lives and they’re busy at different times. So it’s just making sure that it’s going to fit in with the participant’s life and lifestyle. (Participant 001, female, age range 55–64, asthma)

**Table 3:** Key themes identified from patient telephone interviews and HCP advisory board meetings

Trial stage	Patients (*N* = 10)	GPs (*n* = 9)	PMs (*n* = 7)
**Pre-trial experience**	Positive	Positive	Positive
	• Personal health benefit • Supporting science • Access to new treatment • Altruism	• Financial benefits • Trial research nurse team • Patient benefit • Influence of other participating sites • Access to hard-to-reach patients • Sponsor communication/support • Inclusion and exclusion criteria	• Patient benefit • Practice financial benefit • Novel study design • Access hard-to-reach patients • Achieve QOF targets • Elicit large data source
		Negative	Negative
		• Room availability • Recruitment	• Room availability • SLS training • Recruitment • Data collection process
**Experience during the trial**	Positive	Positive	Positive
	• Trial research nurse team • Study assessments • Study location • Free prescriptions	• Expectations met • Negative pre-conceptions dispelled • Patient knowledge of condition • Better quality of care • Study organization • Trial research nurse team • Minimal burden on workload	• Expectations met • Knowledge from practice nurses • Patient health improvement • Study organization • Minimal impact on practice • Reduced practice workload • Sponsor support • Trial research nurse team
	Negative	Negative	Negative
	• Not receiving study treatment • No study results • Non-participating pharmacies	• Prescriptions • eCRF system • Training • Trial staff turnover • Recruitment • Non-participating pharmacies	• Financial burden (e.g. patient reimbursement) • Invoice/prescription system • Trial staff turnover • Transfer and data storage • Room availability • Reporting hospitalizations
**Post-trial experience**	Positive	Positive	Positive
	• Improved knowledge of condition • Involved in a research study • Improved inhaler adherence • Improved symptoms and health	• Treatment effectiveness • Patient treatment satisfaction • Willingness to prescribe study drug • Professional development • Confidence in research	• High degree of satisfaction • Expectations surpassed • Financial benefit invested in the practice
	Negative	Negative	Negative
	• Did not receive COPD PLS	• Unaware of COPD results and COPD PLS • No incentive to share results	• Unaware of COPD results and COPD PLS • Current PLS of low value to patients
**Future trial experience**	Positive	Positive	Positive
	• High likelihood of participating in research • Involvement in design of studies	• High likelihood of participating in research • Confidence in actively seeking research • Involvement in design of studies	• High likelihood of participating in research • Increased confidence to participate • Involvement in design of studies • Role similar with that in SLS
	Negative		Negative
	• Unlikely to actively seek research		• Impact of GDPR implementation • Unlikely to actively seek research

*COPD* chronic obstructive pulmonary disease, *eCRF* electronic case report form, *GDPR* General Data Protection Regulation, *GP* general practitioner, *HCP* healthcare professional, *QOF* Quality Outcomes Framework, *PM* practice manager, *PLS* plain language summary, *SLS* Salford Lung Studies

### GP experience

Key themes from the GP advisory board are summarized in Table 3 (detailed results in Additional Table 2). The primary motivation for GPs’ decision to participate was financial support, which enabled them to allocate time and resources to the SLS without increasing strain on their practices. GPs had a mainly positive pre-trial experience, notably regarding the provision of research nurse support during study set-up and the non-restrictive patient recruitment criteria:

The support [of the study nurses] was fantastic. It was really easy because the nurses did it for us to a large degree. And I think without that, if we had to do it ourselves, the remuneration just wouldn’t’ve made sense. (GP, Principal Investigator)

Negative comments focused on issues with patient recruitment, e.g. non-participating pharmacies:

We had a pharmacy that didn’t take orders. It was one in one of our deprived [areas], on our patch…they weren’t signed up to SLS, so we couldn’t recruit any of those patients that were using that pharmacy. (GP, Principal Investigator)

GPs reported a largely positive experience during the trial. The trial was described as well-organized, with good sponsor support, and inclusion of research nurse support was highlighted as a key benefit. Challenges such as recruiting enough patients and prescription errors were mostly resolved internally at GP practices. Although few, negative comments of the trial included safety reporting via the electronic case report form (eCRF) and staff turnover:

There’s a lot of safety things I felt didn’t need to be reported…I thought it was crazy. I don’t know if they have protocols on what you had to capture. It just seemed a bit OTT [over the top] to me, but maybe that’s what you wanted. (GP, Principal Investigator)

So you are getting used to how you worked with her [research nurse]…and then they come and another one goes, and you are almost learning the system again because she does it the other way. (GP, Principal Investigator)

Post-trial, GPs considered the value of the trial as largely patient-focused, although some highlighted the professional value of, and increasing their confidence in, participating in research:

It’s opened our eyes up a little bit to the possibilities of research as well… We’re not frightened of it. (GP, Principal Investigator)

Despite receiving notifications from the study team, GPs were generally unaware of the trial results. They also did not recall receiving the plain language summary (PLS) of COPD trial findings and were unaware of the plan to disseminate it to patients:

Has there been any mechanism by which plain language summary has been sent to the patients who participated? (GP, Sub-principal Investigator)

All GPs stated that they would be very likely to participate in future trials, but this was dependent on important trial aspects such as a study design similar to the SLS and appropriate financial support. Most important, however, was provision of research nurse support:

As a practice that hasn’t got one [research nurse], it would be very important to us that we had. So extra back-up and support, because it just wouldn’t be viable for them. (GP, Principal Investigator)

### PM experience

Feedback from the PM advisory board is summarized as key themes in Table 3 (detailed results in Additional Table 3)*.* PMs noted that the final decision to participate in the trial was taken by the GPs, but all had been included in discussions:

I remember there was a team from SLS came and spoke to the entire practice…it was an absolute itinerary of everything that would be expected of practices…we were consulted and we were considered and we were allowed time to think about being involved… (PM, female)

While some issues surrounding room resourcing and complex training were raised, PMs considered involvement in a large trial, potential patient benefit and financial benefit to practices as key drivers of participation and the perceived value of the trial:

…so we’d meet the QOF [quality outcomes framework] targets, and so it would help with our annual reviews and management of those patients throughout the year. (PM, male)

PMs reported mostly positive, but some negative, experiences during the trial. They commented that the trial was well-organized, well-supported, and caused minimal disruption:

It was exactly what we thought it would be and exactly what we were told it would be. There was no surprises; it was exactly what it was. The way we were prepped for it by [the sponsor] was excellent. (PM, male, telephone interview)

Like GPs, PMs considered the research nurses instrumental to successful implementation and participation. However, they also commented on the high nursing staff turnover as well as complex and time-consuming invoicing and prescription systems:

The thing is, once it’s [prescription] logged, it’s already logged so you had to ring up somebody to get them to delete the one that had been printed. Then you’d have to physically go back in and reissue it…I get why it was like that, I do, but it was a logistical nightmare. (PM, female)

A minor, but notable challenge experienced by practices during the trial related to the initial financial strain of patient reimbursement on practice finances (all patients in the SLS were reimbursed for their time for activities outside of standard care):

We had a float of £500, I mean, we were only a small practice, smaller group and you were forever having to go and top it up and that was one thing I’ll say, keeping a tally of everything we had paid out, claiming was a bit difficult. (PM, female)

PMs expressed a high degree of post-trial satisfaction, surpassing expectations for many. This included seeing clear benefits of the trial for patients and long-term benefits for the practice, e.g. reinvestment of remuneration:

We did invest some monies because we knew we needed more nursing time in terms of [a] health care assistant to alleviate some of the tasks that the nurses didn’t need to do… That wasn’t as a consequence of the study, that was a bonus, a benefit from the study. (PM, female)

The post-trial dissemination of the SLS results was a minor negative, with most PMs unaware of the published COPD results and COPD PLS. PMs considered the COPD PLS of low value to patients due to its complexity.

Like GPs, PMs expressed a high likelihood of participating in future trials. Key factors in this were research nurse support, and a study design, support and resources similar to the SLS. However, future trials that required the practice to take on roles/responsibilities similar to those that were provided by the SLS support network would not be attractive propositions. PMs noted that the SLS had raised their expectations about participating in future trials. Most PMs indicated interest in providing input on the design of future studies:

We work in general practice. We know what works in general practice and what doesn’t, so, you know, I think it’s quite valuable the insight that we would have. (PM, female)

### Insights on improvements for future effectiveness RCTs

**Fig. 2:**
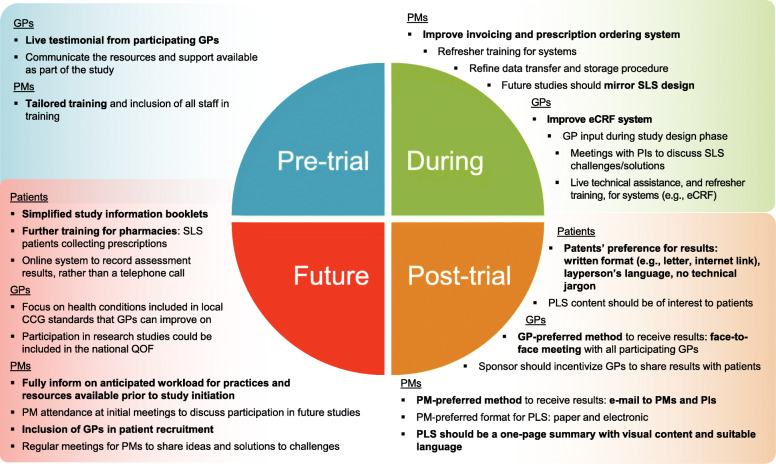
Summary of main findings regarding suggested improvements for future trials. Bold text indicates improvements considered of more importance. Abbreviations: CCG, Clinical Commissioning Group; eCRF, electronic case report form; GP, general practitioner; GSK, GlaxoSmithKline plc.; PI, principal investigator; PLS, plain language summary; PM, practice manager; QOF, Quality Outcomes Framework; SLS, Salford Lung Studies

Patients were asked for suggestions for overall improvement of future trial experience, while GPs and PMs were asked for their suggested improvements across each of the four main trial stages. Key insights are presented in Fig. 2.

For patients, the main areas for improvement were focused around simplifying study information, the inclusion and training of more local pharmacies, and receiving the study results. Suggested improvements from GPs included a local participating GP testimonial and clearer communication of available resources and support (pre-trial stage), regular principal investigator meetings and improvements to the eCRF system (during the trial), and improving the dissemination of study results (post-trial stage). GPs also suggested including studies as part of a national rewards and incentive programme to increase GP engagement in future trials. PMs suggested a variety of improvements, including tailored training for practice staff (pre-trial stage), improved prescription and invoicing systems (during the trial) and improvements to the study PLS (post-trial stage). To improve the overall experience of participation, PMs suggested their inclusion in the initial study meetings, clearer communication of resources and support, and regular PM meetings during the trial.

## Discussion

Our qualitative approach demonstrated an overall positive experience of participating in the SLS for patients and HCPs. All participant groups highlighted the ease and flexibility of the study assessments and the excellent relationships with study research nurses as being key contributors to the acceptability of this effectiveness study. Minor challenges for HCPs related to data reporting, research nurse turnover, invoicing and prescription systems and patient reimbursement processes. All participant groups indicated a high likelihood of participating in a future trial, with HCPs adding provisos regarding research nurse support and a study design comparable to the SLS. Several strategies to encourage trial participation were suggested, such as clearly communicating benefits to patients and ensuring flexible study assessments.

Typically, RCTs compare an intervention with a clearly defined comparator/placebo under ideal study conditions in a homogenous patient population, such that confounders are minimized and rigorous conclusions can be made regarding the efficacy of the intervention [1, 2]. A major drawback of this approach, however, is the limited ability to generalize results to routine clinical practice, where patients and treatment strategies are vastly more varied. In contrast, real-world effectiveness (pragmatic) RCTs test the effectiveness of an intervention in a much less tightly defined setting, with the aim of providing evidence of whether an intervention actually works in routine clinical practice. Advantages of this approach include the applicability of study results to the wider patient population and utility in comparing complex treatment options.

Despite the growing appreciation of real-world effectiveness RCTs, there are limited data describing the experiences of those participating in them. Previous studies have described the patient experience of recruitment to clinical trials, focusing on patients’ decision-making processes in relation to participation, but providing little information on their experiences during the study, their views on future participation or on the value of the study [27]. The findings from this project provide valuable insights from key stakeholders instrumental to the delivery and success of such trials. Trial experiences were collected across a broad range of topics to comprehensively explore the operational and logistical aspects associated with pre-trial, during-trial and post-trial stages of the SLS. To ensure study impartiality, researchers from an independent non-profit research service provider undertook data collection and analysis; this may have allowed participants to more freely express their experiences.

The main limitation of this work was the time elapsed between the end of participants’ roles in the SLS and the initiation of this qualitative research (up to 5 years in some cases), which may have affected recall. While the overall sample size (*n* = 26) was comparable with other qualitative research conducted in similar settings using broadly similar methodologies (analysis of semi-structured interviews, focus groups, interview transcripts) [28–32], the numbers in each participant group were small. The limited sample of patients (*n* = 10) reflected the difficulty and considerable effort required to re-establish contact with patients and GPs from the SLS. Furthermore, a reduction in the planned number of GPs included in the advisory board from 12 to nine was necessitated by the event coinciding with a particularly busy time of year for GPs in the UK. Our sample comprised only volunteers; those with a poor experience of the SLS may have been less likely to volunteer. The SLS were conducted in a single region in North West England; thus, the socioeconomic spread of our sample was limited, which may affect generalizability. However, this is mitigated by our focus on features of effectiveness trial design and delivery, which are not location-dependent.

There is very limited literature describing the drivers for patient and HCP engagement in effectiveness trials; therefore, this work provides unique and important insights. Consistent with previous research [6], altruism and potential for personal benefits were cited by participants as contributors to their participation in the SLS. Similar to other studies [8, 11, 14, 33], HCPs identified the importance of time and resources required for participation in clinical trials. Our findings add to current knowledge by identifying the provision of good quality research nurse support as essential, both prior to and during the trial. A notable feature of the SLS effectiveness trials, particularly highlighted by PMs, was the minimal disruption to the day-to-day running of practices. Minimizing time commitments, administrative burden and potential conflict with regular clinical duties is important, since these are known barriers to research participation [9, 34].

Clearly communicating the value and benefits of trials to patients and HCPs at an early stage is recommended to enhance engagement and participation [35]. The importance of this is illustrated by the observation in this study that patients, GPs and PMs from the SLS all expressed this sentiment as ways in which future trial participation and experience may potentially be improved.

To provide data on effectiveness in conditions closer to routine practice, assessment of outcomes in effectiveness RCTs should interfere as little as possible with standard care [26]. Patients in the SLS commented positively on the ease and convenience of the study assessments, indicating that the trials were successful in this context.

Previous work shows that study participants are very receptive to receiving trial results as aggregate and clinically significant individual results from the trial in which they have participated [36, 37]. Indeed, sponsors are required to provide summary results within 1 year of trial end [38]. As required, disseminating the trial results was a feature of the SLS design, although some patients commented on not receiving the COPD results and GPs and PMs were largely unaware of the results and their role in the dissemination of these. Thus, even with dedicated strategies for dissemination, additional efforts may be required in future trials to ensure the successful dissemination of trial results to patients and HCPs alike.

Patient and HCP engagement and participation in clinical research trials are influenced by a variety of factors that can make recruitment challenging. The success of the effectiveness study approach taken in the SLS is highlighted by patients’ positive feedback around the simplicity and convenience of the study assessments, which importantly also extended to the experiences of GPs and PMs, who reported minimal disruption to practice day-to-day operations. Moreover, all patients, GPs and PMs who participated in this research reported that they would very likely participate in future trials. This would depend on simple and convenient study assessments from the patient perspective, while the provision of research nurse support was considered essential to the future participation of GPs and PMs. It is notable that patients considered that the benefits of participation, and for GPs and PMs the resources and support available in the trial, need to be more clearly communicated at the recruitment stage. Future effectiveness RCTs could be improved by simplifying study information for patients and ensuring that HCPs are provided with relevant training and have access to user-friendly study systems. Patients and HCPs are also clearly interested in contributing to trial design, a finding noteworthy for sponsors.

## Conclusions

Overall, the experiences of patients and of GPs and PMs show that the overall objectives of the design and delivery of SLS, i.e. minimal disruption to normal care and minimal burden to patients, were successfully met. The experiences of patients and HCPs in the SLS provide valuable insights that will help inform future best practice in the design and conduct of future real-world effectiveness RCTs in primary care. The detailed first-hand experiences of HCPs reported here are a valuable source of information that will be of significant interest to others considering engaging with clinical research and participating in effectiveness RCTs.

## Supplementary information

**Additional file 1: Table 1.** Summary of key findings from the patient telephone interviews. Table 2 Summary of key findings from the GP advisory board meeting. Table 3 Summary of key findings from the PM advisory board meeting.

## Data Availability

The anonymized qualitative datasets analysed during the current study and associated documentation may be requested by submitting an enquiry via www.clinicalstudydatarequest.com.

## Abbreviations

COPD: Chronic obstructive pulmonary disease
eCRF: Electronic case report form
GDPR: General Data Protection Regulation
GP: General practitioner
HCP: Healthcare professional
IMD: Index of Multiple Deprivation
PLS: Plain language summary
PM: Practice manager
QOF: Quality Outcomes Framework
RCT: Randomized controlled trial
RTI-HS: RTI Health Solutions
SLS: Salford Lung Studies
