# Supplementary material for: Patient and healthcare professional experiences of the Salford Lung Studies: qualitative insights for future effectiveness trials
Source: Trials. 2020 Sep 17;21:798. doi: 10.1186/s13063-020-04655-x (PMC7499906; doi:10.1186/s13063-020-04655-x)
Supplement: Supplementary file 1 — Additional file 1: Table 1. Summary of key findings from the patient telephone interviews. Table 2 Summary of key findings from the GP advisory board meeting. Table 3 Summary of key findings from the PM advisory board meeting. [file 13063_2020_4655_MOESM1_ESM.docx]

**Additional material**

**Additional Table 1** Summary of key findings from the patient telephone interviews

| **Pre-trial experience** | - Patients’ rationale for participation was multifaceted. For some, the primary motivation was personal health benefits - The study information was considered clear and detailed enough to allow patients to make an informed decision - Only minor negative comments were reported in relation to the length and content of the study information |
| --- | --- |
| **Experience during the trial** | - Patient-reported experience during the study was mainly positive - Key features included the convenience and ease of the study assessments, the positive relationships with the research nurses, and receiving free prescriptions during the study |
| **Post-trial experience** | - Patient-perceived general value of the SLS included the improvement of general COPD and asthma knowledge and the potential benefit for other people - Patient-perceived personal value of the SLS focused on improving knowledge of their condition and symptoms - Six patients were interested in the results from the SLS. Patients preferred receiving the results via a letter, an internet link, or a face-to-face meeting. Patients noted that results should be written in layperson’s language, no technical jargon - The need for an improved strategy to disseminate results to patients |
| **Future trial experience** | - All patients reported that they would participate in a future study, and most would encourage other people to participate in research studies. Participation would be dependent on several factors, including the study logistics, disease severity, the risks of participation, and the study design - Strategies to encourage patient participation included the clear communication of benefits associated with participation, flexible study assessments, and an appropriate financial incentive |

Abbreviations:
COPD: chronic obstructive pulmonary disease; SLS: Salford Lung Studies.

**Additional Table 2** Summary of key findings from the GP advisory board meeting

| **Pre-trial experience** | - Financial support was a primary motivation for GP participation as this enabled GPs to participate in the SLS without increasing the strain on their limited resources - The experience of the pre-trial phase was mainly positive, particularly the support from the study nurses and the non-restrictive inclusion and exclusion criteria that facilitated patient recruitment - The negative pre-trial experiences focused on challenges to recruitment associated with non-participating pharmacies and non-English–speaking patients |
| --- | --- |
| **Experience during the trial** | - The support provided by the study nurses and their relationship with GP site staff members and patients was highlighted as a particularly positive experience of the SLS - The negative aspects of the SLS comprised ordering medications, reporting safety data via the eCRF system, and staff turnover rates |
| **Post-trial experience** | - GP-perceived value of the SLS was predominantly patient focused - GPs were generally unaware of the published study results. Only a few GPs recalled receiving the results - Many GPs were also unaware of the COPD PLS and the intention that GPs would disseminate the results to their patients. GPs were concerned that they lacked the time and/or resources necessary to share the results with patients |
| **Future trial experience** | - GPs expressed a high likelihood of participating in future studies. However, participation would be dependent on several factors, including suitable financial support, the inclusion of a research nurse, and a similar study design to the SLS |

Abbreviations:
COPD: chronic obstructive pulmonary disease; eCRF: electronic case report form; GP: general practitioner; PLS: plain language summary; SLS: Salford Lung Studies.

**Additional Table 3** Summary of key findings from the PM advisory board meeting

| **Pre-trial experience** | - All PMs had been included in the meetings with GPs to discuss the SLS - Perceived drivers for participation in the SLS included financial benefits (e.g. savings made on the prescription budget), benefits associated with meeting QOF targets, benefits to patients (e.g. self-management of COPD, access to free inhalers), and taking part in a novel study exclusive to Salford - Availability of rooms was the primary concern for PMs during the initial study set-up - Training was noted as burdensome and complex |
| --- | --- |
| **Experience during the trial** | - PMs noted the SLS was well-organized and caused minimal disruption to the day-to-day operations in the GP practices - The most notable challenge was the financial burden of the SLS, particularly the payment of patient reimbursement - The invoice system was reported as burdensome, often requiring assistance from the sponsor to complete invoices - The prescription system was highlighted as cumbersome and not user friendly - Reporting hospitalisations within 24 hours was deemed difficult by one PM whose practice was on the periphery of the Salford area and therefore not part of the integrated care record system |
| **Post-trial experience** | - The PMs expressed a high degree of satisfaction with the study, particularly as they deemed it to have been a success - The SLS provided GP practices with long-term benefits such as the reinvestment of the remuneration received for participating in the study, including purchasing new equipment and furniture, offering extra work hours to staff, and providing opportunities for staff professional development - PMs were unaware of the results from the study or the COPD PLS. The review of the PLS identified several issues, including length, content, and layout. PMs would prefer a less complex, one-page PLS with more visuals, containing results of interest to patients (e.g. patient satisfaction with the trial drug and not exacerbation rates) |
| **Future trial experience** | - PMs expressed a high likelihood of participating in future studies. However, participation would be dependent on the similarity of the study to the SLS and the inclusion of a study nurse - PMs expressed concerns regarding initial contact of patients for future studies due to recent implementation of GDPR - Successful implementation of the SLS provided PMs with confidence regarding research participation |

Abbreviations:

COPD: chronic obstructive pulmonary disease; GDPR: General Data Protection Regulation; GP: general practitioner; QOF: Quality Outcomes Framework; PLS: plain language summary; PM: practice manager; SLS: Salford Lung Studies.
